# An Efficient and Self-Adapting Localization in Static Wireless Sensor Networks

**DOI:** 10.3390/s90806150

**Published:** 2009-08-04

**Authors:** Guodong Teng, Kougen Zheng, Wei Dong

**Affiliations:** 1 College of Computer Science, Zhejiang University, Hangzhou, 310027, China; E-Mails: teng@zju.edu.cn (G. T.); dongw@zju.edu.cn (D. W.); 2 Hangzhou Normal University, Hangzhou, 310036, China

**Keywords:** Wireless Sensor Networks (WSNs), localization, particle filter, Self-Adapting Mobile Beacon-assisted Localization (SA-MBL)

## Abstract

Localization is one of the most important subjects in Wireless Sensor Networks (WSNs). To reduce the number of beacons and adopt probabilistic methods, some particle filter-based mobile beacon-assisted localization approaches have been proposed, such as Mobile Beacon-assisted Localization (MBL), Adapting MBL (A-MBL), and the method proposed by Hang *et al*. Some new significant problems arise in these approaches, however. The first question is which probability distribution should be selected as the dynamic model in the prediction stage. The second is whether the unknown node adopts neighbors’ observation in the update stage. The third is how to find a self-adapting mechanism to achieve more flexibility in the adapting stage. In this paper, we give the theoretical analysis and experimental evaluations to suggest which probability distribution in the dynamic model should be adopted to improve the efficiency in the prediction stage. We also give the condition for whether the unknown node should use the observations from its neighbors to improve the accuracy. Finally, we propose a Self-Adapting Mobile Beacon-assisted Localization (SA-MBL) approach to achieve more flexibility and achieve almost the same performance with A-MBL.

## 1. Introduction

Recent advancements in wireless communications and electronics have enabled the development of low-cost sensor networks [1]. There are several essential issues (e.g., localization, deployment, and coverage) in Wireless Sensor Networks (WSNs). Localization is one of the most important subjects because the location information is typically usefully for coverage, deployment, routing, location service, target tracking, and rescues [2].

The localization of static WSNs relies on several beacons which know their locations scattered throughout the sensor networks and the precision of the localization increases with the number of beacons [3]. To reduce the number of beacons, many mobile beacon-assisted approaches have been proposed. These approaches can be categorized based on whether the localization techniques are *Range-based* or *Range-free*, whether the localization algorithms are *Centralized* or *Distributed*, and whether the localization results are *Deterministic* or *Probabilistic*.

Compared with previous works on mobile beacon-assisted localization [4–15], Mobile Beacon-assisted Localization (MBL) [3] integrates many more of the advantages of the methods, such as range-free technique, distributed algorithm and probabilistic approach. Paper [3] also proposes another localization approach based on MBL, called Adapting MBL (A-MBL), to increase the efficiency and accuracy of MBL by adapting the size of sample sets and the parameter of the dynamic model during the estimation process. Evaluation results show that the accuracy of these approaches outperforms some other approaches [13,16] when both of them use only a single mobile beacon for localization in static WSNs. Some new significant problems arise about A-MBL, however.

### Dynamic model

In general, the prediction stage in the particle filters uses the dynamic model to predict the state probability density forward from one measurement time to the next. Particularly in particle filters based mobile beacon-assisted localization, the dynamic model is needed for unknown nodes to provide enough variability in choosing new samples, i.e., which is used to limit a sample impoverishment phenomenon. MBL (or A-MBL) and Hang *et al.* [14] adopt uniform distribution and normal distribution as the dynamic model, respectively. To the best of our knowledge, no theoretical analysis or experimental evaluations are given to explain why this particular dynamic model should be adopted, however. In fact, on one hand, the choice of the dynamic model will affect the accuracy of localization results. On the other hand, the approach to simulating random variables of the dynamic model will affect the efficiency of localization process. To this end, an appropriate dynamic model should improve the location accuracy when locating unknown nodes and time efficiency when simulating random variables.

### Neighbors’ observation

In general, the update operation uses the latest measurement to modify the prediction probability density. Particularly in particle filters based mobile beacon-assisted localization, the unknown node filters the impossible samples based on new observations. MBL (or A-MBL) only relies on the observations from the beacon. This has two advantages. First, the number of unknown nodes will not affect the accuracy of localization. Second, the computation and communication costs drop drastically, since nodes are no longer involved in the localization of other nodes [16]. Typically, network protocols maintain information about their neighbors in order to make informed decisions for routing, aggregation, and dissemination [17]. Some proposed approaches, such as Mobile and Static sensor network Localization (MSL) [16], use observations from the neighbors of unknown nodes, which may have greater communication costs, to increase efficiency and accuracy. It is still unknown whether or not to use observations from the neighbors in mobile beacon-assisted approaches, however. It is known that adopting neighbors’ observation should improve the location accuracy when locating unknown nodes and communication efficiency when collecting observations.

### Adapting mechanism

The parameters in the dynamic model will affect the accuracy and efficiency of particle filter-based mobile beacon-assisted localization. As an example concerning the number of samples [3,16,18], under the same conditions, keeping more samples improves efficiency at the beginning of localization. The time complexity of the update stage and the memory requirements to keep samples are both linear in the number of samples needed for the estimation, however. Another example about the parameter *α* in the dynamic model [3,18], a greater value of parameter in the dynamic model improves the efficiency at the beginning of localization and the accuracy at the other extreme. Though adopting predefined adjustment tables in A-MBL is convenient and effective, obtaining these tables is difficult. To this end, the key question in A-MBL is how to determine the number of samples and the value of parameter in the dynamic model to achieve more flexibility. It is known that the approach should be unrelated to the deployment region, the location time and the number of unknown nodes, i.e., the approach should be self-adapting.

To address above problems, we first describe three different dynamic models and some related approaches to simulating random variables of the dynamic models. The theoretical analysis and experimental evaluation will be given to explain why the particular dynamic model should be adopted. Then, we describe how to make use of the neighbors’ observations in the update stage. Next, we give the condition whether the unknown node use the observation from the neighbors to improve the accuracy. Finally, we analysis and compare some approaches to judge the localization to reach the stable phase. As a result, a self-adapting mechanism will be proposed.

Major contributions of this paper are as follows:

- In order to improve the time efficiency in the prediction stage of particle filter-based mobile beacon-assisted localization, we give the theoretical analysis and experimental evaluations to suggest which probability distribution should be adopted in the dynamic model.
- We give the condition for whether the unknown node should use the observation from its neighbors to improve the accuracy.
- We propose a Self-Adapting Mobile Beacon-assisted Localization (SA-MBL) approach to achieve more flexibility and obtain almost the same performance as with A-MBL.

The rest of this paper is organized as follows: Section 2 describes the algorithm of A-MBL. Section 3 analyses and discusses the different dynamic models, the neighbors’ observation, and the self-adapting mechanism. Section 4 shows and discusses our evaluation results. Section 5 gives an overview of related works. Finally, Section 6 concludes our work.

## 2. Related Work

Much research has been done on mobile-beacon assisted localization for WSNs. A general survey can be found in [3]. Here, we provide a brief survey focusing only on Monte Carlo localization techniques suitable for static or dynamic WSNs. The range-free, distributed and probabilistic algorithm MCL [18] proposed by Hu *et al.* only works in mobile sensor networks. In the prediction phase, MCL applies the mobility model to each sample to obtain a set of new samples. The probability of current location based on previous location estimates is given by a uniform distribution.

Different from MCL, in the range-free, distributed and probabilistic algorithms MSL and MSL^*^, each sensor node uses observations from only those neighbors that have better location estimates that it, i.e., the weight of a sample is determined using the neighbors’ observations besides the location announcements from the beacons. MSL modified the mobility model to allow this algorithm to work in static WSNs. As in the MCL, the uniform distribution is adopted as the mobility model in MSL.

In addition, some Monte Carlo localization specially designed for static sensor networks have been proposed. Hang *e*t *al*. in [14], discuss a hybrid (range-free and range-base), centralized and probabilistic algorithms in the context of the static WSNs localization using a single mobile beacon. This algorithm assigns a dynamic model to enable samples to move closer to the actual location, and the dynamic model adopts a normal distribution. After the location distribution of unknown node is update, the beacon will also update the location distribution of the unknown node’s neighbors based on the one-hop observation data between the pair of neighbors. MA-MCL [15] does a similar work which proposes a range-free, centralized and probabilistic localization approach. MA-MCL adopts a uniform distribution as the mobility model.

In [3], we proposed a range-free, distributed and probabilistic MBL approach. This approach outperforms both MSL and Arrival and Departure Overlap (ADO) [13] when both of them use only a single mobile beacon for localization in static WSNs. In the prediction phase, like MCL and MSL, MBL adopts a uniform distribution as the dynamic model. This paper also proposes A-MBL, to increase the efficiency and accuracy of MBL by adapting the size of sample sets and the parameter of the dynamic model during the estimation process. As the MBL, A-MBL does not use the observation information from neighbors.

## 3. Description of A-MBL

### 3.1. Assumption

Similar to the assumption in [3], let us consider a sensor network with *M* static sensor nodes in a 2D plane which do not have *a priori* known locations (called *unknown nodes*) and a single mobile node (called *beacon*), equipped with localization hardware, e.g., a GPS, which allows it to know its location at all times. We assume that all unknown nodes are randomly deployed in an area of size *S* and each sensor (unknown node or beacon) has the same ideal radio range *r*. The beacon is capable of moving a distance in a time step (*v_b_*) in any direction where 0 ≤ *v_b_* ≤*v_max_*. The beacon knows *v_max_*, but it does not know the value of *v_b_* or the direction of movement in any time step. At time *t*, every unknown node within the radio range of the beacon will hear a location announcement from that beacon. We do not assume very tightly synchronized clocks. In a realistic deployment, it would be necessary to deal with network collisions and account for missed messages [18].

### 3.2. A-MBL

A-MBL uses the *particle filter* approach (also called *sequential Monte Carlo method*) to perform Bayesian filter based localization on a sample representation. The key idea is to represent the required posterior density by a set of random samples with associated weights and to compute estimates based on these samples and weights [19].

Let $\left\{<l_{t}^{i},\;w_{t}^{i}>,\;i=1,\ldots ,N\right\}$ denotes a *random measure* that characterizes the posterior density *p*(*l_t_* | *o_t_*) which denotes the current location estimate *l_t_* conditioned on the observation *o_t_* from the beacon at time *t*, where $L_{t}=\left\{l_{t}^{i},\;i=1,\ldots ,N\right\}$ denotes a set of support *samples* (or called *particles*) with associated *weights* $W_{t}=\left\{w_{t}^{i},\;i=1,\ldots ,N\right\}$ at time *t* and *N* denotes the number of samples of an unknown node.

The details of the A-MBL are as follows:

**Initialization**: In this stage, all unknown nodes have no information about their locations. The initial set of samples Equation 1 for each unknown node is chosen randomly from the whole deployed area and represented by a set of uniformly distributed samples with equal weights Equation 2. The weight equal to one represents the importance of corresponding sample, which infers one of the location estimates of the unknown node:

(1) $$L_{0}=\left\{l_{0}^{i}|l_{0}^{i}∼p\left(l_{0}\right)=\frac{1}{S},\;i=1,\ldots ,N\right\}$$

(2) $$w_{0}^{i}=1,\;i=1,\ldots ,N$$

where *L*_0_ denotes the initial set of each sample, *p*(*l*_0_) denotes initial location probability density, the symbol ∼ denotes sample generated sign, i.e., the samples on the left side are generated from the probability density on the right side, and $w_{0}^{i}$ denotes the initial weight of each sample.

**Prediction**: In this stage, we adopt a *dynamic model* in which the unknown node is capable of moving a distance in a time step (*v_node_*) in any direction where 0 ≤ *v_node_* ≤ α. The unknown node knows α, but it does not know the value of *v_node_* or the direction of movement in any time step. Then, the unknown node generates new samples as follows:

(3) $$P_{t}=\left\{l_{t}^{i}|l_{t}^{i}\;\text{is selected from}\;p\left(l_{t}^{i}|l_{t-1}^{i}\right),\;\text{where}\;l_{t-1}^{i}\in L_{t-1}\;\text{for all}\;\;i=1,\ldots ,N\right\}$$

where *P_t_* represents the approximation of prior density at time *t* after the prediction stage, *L*_*t−1*_ represents the approximation of posterior density at time *t−1*, and the *transition equation* for each sample described as follows:

(4) $$p\left(l_{t}|l_{t-1}\right)=\{\begin{array}{ll} \frac{1}{\pi \alpha^{2}} & \mathrm{if}\;\;\mathrm{filter}(R)=\mathrm{TRUE}\\ 0 & \mathrm{if}\;\;\mathrm{filter}(R)=\mathrm{FALSE} \end{array}$$

where *filter(R)* denotes the unknown node receives the current location announcement of the beacon, but did not receive the location announcement from the beacon’s previous location, or the unknown node received the preceding location announcement from the beacon, but does not receive the location announcement from the beacon’s current location. For more details about *filter(R)* see [3].

**Update**: In this stage, the unknown node filters the impossible samples based on new observations. The unknown node updates samples as follows:

(5) $$U_{t}=\left\{l_{t}^{i}|l_{t}^{i}\;\text{where}\;l_{t}^{i}\in P_{t}\;\;\;\text{and}\;\;\;w\left(l_{t}^{i}\right)=1\right\}$$

where *U_t_* represents the approximation of posterior density at time *t* after the update stage, and the weight $w\left(l_{t}^{i}\right)$ will be obtained by $p\left(o_{t}|l_{t}^{i}\right)$. The weight of sample is determined by the filter condition:

(6) $$w_{t}=p\left(o_{t}|l_{t}\right)=\{\begin{array}{ll} 1 & \mathrm{if}\;\;\mathrm{filter}(R)=\mathrm{TRUE}\\ 0 & \mathrm{if}\;\;\mathrm{filter}(R)=\mathrm{FALSE} \end{array}$$

**Resampling:** A-MBL adopts a Systematic resampling algorithm [20] in this paper since it is simple to implement, takes *O(N_s_)* time, and minimizes the Monte Carlo variation.

**Adapting**: A-MBL adopts two predefined adjustment tables, one for the number of samples *N*, and the other for the parameter α. Once some record in the table is matched, the number of samples and the value of *α* in the unknown node will be adjusted according to the corresponding time.

The complete A-MBL for every unknown node is shown in [3].

## 4. Key Issues

### 4.1. Dynamic Model

In this section, we use the term “*random numbers*” to mean independent *random variables* from a probability distribution, i.e., we use random numbers to simulate random variables from some probability distribution. For example, let random variable Z is uniformly distributed over (0, 1). If U is uniformly distributed over (0, 1) random numbers, then Equation 7 denotes random variables Z are simulated by random numbers U:

(7) Z=U

We denote the horizontal X and vertical Y location of the *i*-th sample of an unknown node at time *t* by $l_{t}^{i}=(X,Y)$.

#### 4.1.1. Square uniform distribution

If we adopt Square Uniform Distribution (SUD) as the dynamic model, i.e., a new sample is generated from each current sample by randomly choosing a point within a square with a side length 2α when the *filter(R)* is equal to *TRUE*. Thus, the transition equation for each sample described as follows:

(8) $$p\left(l_{t}|l_{t-1}\right)=\{\begin{array}{ll} \frac{1}{4\alpha^{2}} & \mathrm{if}\;\;\mathrm{filter}(R)=\mathrm{TRUE}\\ 0 & \mathrm{if}\;\;\mathrm{filter}(R)=\mathrm{FALSE} \end{array}$$

Next, we simulate SUD from random numbers shown as follows:

Let the horizontal *X* and vertical *Y* location of the *i*-th sample of an unknown node are independent uniform random variables on the interval (−*α*, *α*). If *U*_1_ and *U*_2_ are independent uniformly distributed over (0, 1) random numbers, then

(9) $$\begin{array}{l} X=-\alpha +2\alpha *U_{1}\\ Y=-\alpha +2\alpha *U_{2} \end{array}$$

Each unknown node will use Equation 9 to generate its own samples when SUD is adopted as the dynamic model.

#### 4.1.2. Circle uniform distribution

If we adopt Circle Uniform Distribution (CUD) as the dynamic model, i.e., a new sample is generated from each current sample by randomly choosing a point within a circle centered at the current location of the sample and the radius α when the *filter(R)* equal to *TRUE.* Then, the transition equation for each sample described as follows:

(10) $$p\left(l_{t}|l_{t-1}\right)=\{\begin{array}{ll} \frac{1}{\pi \alpha^{2}} & \mathrm{if}\;\;\mathrm{filter}(R)=\mathrm{TRUE}\\ 0 & \mathrm{if}\;\;\mathrm{filter}(R)=\mathrm{FALSE} \end{array}$$

How can the uniform distribution of filters in a circle be realized? One solution could be to generate the filters uniformly in a circle and then draw again if the point is not within the circle. The approach called *Accept-Reject* is shown in Algorithm 1

**Algorithm 1.** Accept-Reject method.

1: **do**
2: Generate random numbers U1 and U2;
3: *X* = −*α* + 2α*U_1_, *Y =* −*α +* 2α*U_2_;
4: **While***X*^2^ + *Y*^2^ ≥ *r*^2^
5: Accept X, Y;

Since the probability that a random point in the square will fall within the circle is equal to π/4 (the area of the circle divided by the area of the square), it follows that, on average, the Accept-Reject method will require 4/π = 1.273 iterations of step 2. Hence, it will, on average, require 2.546 random numbers to generate 2 independent random variables.

A more efficient solution is to choose the angle uniformly as before, but for the radius an intermediate value *U* is generated uniformly between 0 and 1, and then *r* is calculated as r=sqrt(U)*α, where *sqrt*() denotes square root function. The approach is called *polar* method.

**Figure 1.**
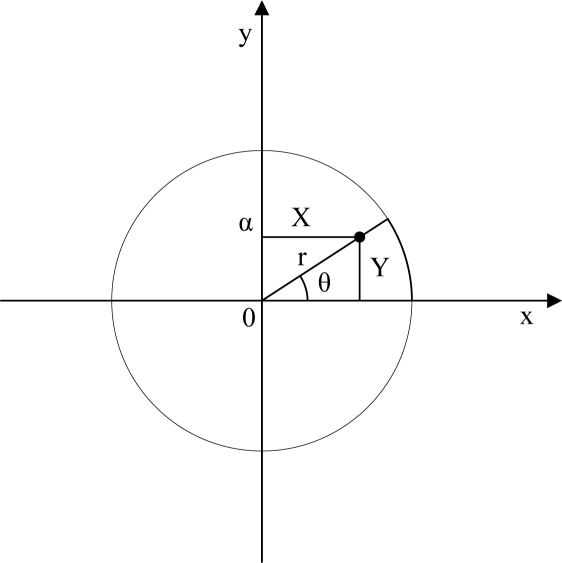
Polar method of CUD.

Formally, let the distribution of samples in a circle of the radius (0, α) with the angle *θ* uniformly between (0, 2π). If *U*_1_ and *U*_2_ are independent uniformly distributed over (0, 1) random numbers, then:

(11) $$\begin{array}{l} \theta =0+(2\pi -0)*U_{1}\\ r=\mathrm{sqrt}\left(U_{2}\right)*\alpha \\ X=r\;\text{cos}\;\theta \\ Y=r\;\text{sin}\;\theta \end{array}$$

where θ is described in Figure 1.

Each unknown node can use Algorithm 1 or Equation 11 to generate its own samples when CUD is adopted as the dynamic model.

#### 4.1.3. Normal distribution

If we adopt Normal Distribution (ND) with parameters 0 and σ^2^ as the dynamic model, then the transition equation for each sample is described as follows:

(12) $$p\left(l_{t}|l_{t-1}\right)=\{\begin{array}{ll} N\left(0,\;\sigma^{2}\right) & \mathrm{if}\;\;\mathrm{filter}(R)=\mathrm{TRUE}\\ 0 & \mathrm{if}\;\;\mathrm{filter}(R)=\mathrm{FALSE} \end{array}$$

Special techniques have been devised to simulate from most of the common continuous distributions [21]. Now, we review the two most popular generating approaches of ND.

(1) Box-Muller

Let the horizontal X and vertical Y location of the *i*-th sample of an unknown node are independent standard normal random variables. If *U*_1_ and *U*_2_ are independent uniformly distributed over (0, 1) random numbers, then:

(13) $$\begin{array}{l} X={\left(-2\text{log}\;U_{1}\right)}^{1/2}\;\text{cos}\left(2\pi U_{2}\right)\\ Y={\left(-2\text{log}\;U_{1}\right)}^{1/2}\;\text{sin}\left(2\pi U_{2}\right). \end{array}$$

The preceding approach to generating standard normal random variables is called the *Box-Muller* method. Its efficiency suffers somewhat from its need to compute the preceding sine and cosine values. There is, however, a way to get around this potentially time-consuming difficulty.

(2) Polar method

The literature [21] also introduces the following approach to generating a pair of independent standard normals:

**Algorithm 2.** Polar method

1: **do**
2: Generate random numbers U1 and U2;
3: Set *V*_1_ = 2*U*_1_ −1, *V*_2_ = 2*U*_2_ −1, $S=V_{1}^{2}+V_{2}^{2}$;
4: **While***S* > 1
5: $X=\sqrt{\frac{-2\;\text{log}\;S}{S}}V_{1},\;Y=\sqrt{\frac{-2\;\text{log}\;S}{S}}V_{2};$

The preceding is called the *polar* method. It will, on average, require 2.546 random numbers, one logarithm, one square root, one division, and 9.546 multiplications to generate two independent standard normals.

If the normal random variable has mean *u* and the variance σ^2^, then it is given as:

(14) $$\begin{array}{l} u+\sigma X\\ u+\sigma Y \end{array}$$

To compare the efficiency of the various algorithms mentioned above, we show the results of the comparison in Table 1. As shown in Table 1, the method to simulate SUD is the most efficient among these algorithms. Whether or not to use SUD as the dynamic model also depends on the accuracy of localization results, however. We will evaluate the localization accuracy of these approaches adopted as the dynamic model in Section 4.2.

### 4.2. Neighbors’ Observation

The aim of combined with the observation from the neighbors is to improve performance, and then get results in faster convergence of the algorithms. To this end, we modify the sampling procedure in MBL to use observations from the neighbors, i.e., each unknown node not only relies on observations from the beacon, but also from the neighbors that have better estimates.

As the approaches described in [16], after choosing a sample from the unknown node, its weight is determined using the beacon and neighbors’ observation. The weight of a sample *l^i^* chosen for the unknown node *p*, is described as *w_l^i^_* (*p*), which is computed as follows: corresponding to the *k-*th neighbor *q_k_* of the unknown node *p*, we set a *partial weight* for the sample *l^i^*, *w′_l^i^_* (*q_k_*). The weight *w*_*l*^*i*^_ (*p*) of sample *l^i^* is the product of the partial weights *w′*_*l*^*i*^_ (*q_k_*) obtained corresponding to each neighbor *q* of the unknown node *p*. That is:

(15) $$w_{l^{i}}\;(p)=\prod_{k=1}^{n}w_{l^{i}}^{\prime }\;(q_{k})$$

where *n* is the number of neighbors of the unknown node *p*.

The partial weights *w′*_*l*^*i*^_ (*q_k_*) of the sample *l^i^* corresponding to observations from the beacon or the neighbors of unknown node are computed as follows:

1. If the unknown node receives the observation from the beacon, then the partial weight *w′*_*l*^*i*^_ (*q_k_*) of sample *l^i^* will be computed by the Equation 6 as MBL (or A-MBL).
2. If the unknown node receives the observations from its neighbors, then the partial weight *w′*_*l*^*i*^_ (*q_k_*) of the sample *l^i^* corresponding to the neighbor *q_k_* is computed using the weights $w\left(q_{k}^{j}\right)$ of the sample $q_{k}^{j}$ of the neighbor *q_k_* as follows:
  (16) $$w_{l^{i}}^{\prime }\;\left(q_{k}\right)=\sum_{j=1}^{N}w\left(q_{k}^{j}\right),\;\;\;\;\mathrm{where}\;\;\;d\left(l^{i},\;q_{k}^{j}\right)\leq r$$

**Figure 2.**
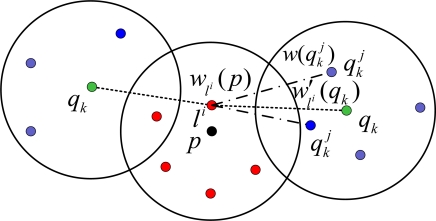
The weight of neighbors.

To summarize the inter-dependencies of the weights amongst the relevant node, we visualize the observations in Figure 2. In Figure 2, the black dot denotes the current unknown node and the red dots denote the samples of it. The green dots denote the neighbor of the unknown node and the blue dots denote the samples of neighbors. The dotted lines between the red dot and green dot denote the partial weight *w′*_*l*^*i*^_ (*q_k_*) of sample *l^i^*. The dash dot line between the red dot and the blue dot denote the weights $w\left(q_{k}^{j}\right)$ of the sample $q_{k}^{j}$ of the neighbor *q_k_*.

The sample *l^i^* is kept if *w*_*l*^*i*^_ (*p*) is greater than a threshold value *β*.

An important question is that the right conditions should be met in order to combine with the observation from neighbors is to improve accuracy and obtain faster convergence of the algorithms.

Nagpal *et al.* [22] have claimed that π*r*/4*n_d_* is a lower bound for the error in any range-free localization algorithm in static sensor networks, where *n_d_* is the node density, the average number of nodes in one hop transmission range, i.e., *n_d_* is the average number of neighbors to an unknown node. In our scenarios, using a single mobile beacon that knows its position is broadly equivalent to using many static beacons each broadcasting once. We may consider each observation from mobile beacon to an unknown node as one constraint (bounded) from the static beacon in the radio range of the unknown node. Thus, the number of observations to the unknown node in mobile beacon-assisted approach is equal to the number of constraints from static neighbor beacons of the unknown node, i.e., the number of observations in mobile beacon-assisted approach is equal the node density, *n_d_*. As a result, if the number of observations from the mobile beacon to the unknown node is larger than *n_d_*, the neighbors’ observation will not improve the precision of the unknown nodes.

### 4.3. Self-Adapting Mechanism

How to determine the number of samples and the value of parameter *α* in the dynamic model to achieve more flexibility is the key question to A-MBL. On the one hand, likelihood-based adaptation [23], KLD-Sampling adaptation [24], and coefficient of variation [3] do not judge the accuracy of MBL to reach the stable phase. On the other hand, the values in such predefined adjustment tables in A-MBL are related to the localization time, i.e., different scale deployment of the sensor nodes requires different predefined adjustment tables. Obtaining these tables is hard work. The intuitive answer to those questions is that MBL needs a self-adapting mechanism to achieve more flexibility, and the corresponding approach is called Self-Adapting MBL (SA-MBL).

The following major factors should be considered to satisfy the self-adapting mechanism:

1. The approach should judge the localization to reach the stable phase as the effect of pre-defined table in A-MBL.
2. The approach should be unrelated to the scale deployment of sensor nodes, the localization time, and the speed of the beacon, etc., but just related to the unknown nodes themselves.

In other words, in order to better judge the stable phase of localization, we need some stable attribute obtained from the unknown nodes themselves.

We know that the locations of samples are in continuous convergence as new observations are incorporated in the initialization phase. When the accuracy of MBL reaches the stable phase, the distribution of samples will also reach a stable phase. To this end, we hope to find the statistical distribution of samples which can be closer to above-mentioned objectives.

We suppose that the samples of an unknown node are sampling from a SUD when we adopt the SUD as the dynamic model. Then, we want to obtain the parameters in the SUD of the samples when the localization reaches the stable phase. We adopt the Method of Moments Estimators (MME) to estimate the parameter of the SUD.

To a single unknown node, the horizontal *X* and vertical *Y* location of the *i*-th sample have two unknown parameters, respectively. Let the horizontal location X of the *i*-th sample have two unknown parameters [*a, b*]. *X^1^*, *X^2^*,…,*X^N^* are horizontal locations of samples *l^1^*, *l^2^*,…,*l^N^* of size *N*, respectively. Then, the MME for *a* and *b* is as follows:

(17) $$\begin{array}{l} a=\bar{X}-\sqrt{\frac{3}{N}\;\sum_{i=1}^{N}{\left(X^{i}-\bar{X}\right)}^{2}}\\ b=\bar{X}+\sqrt{\frac{3}{N}\;\sum_{i=1}^{N}{\left(X^{i}-\bar{X}\right)}^{2}} \end{array}$$

where:

(18) $$\bar{X}=\sum_{i=1}^{N}X^{i}$$

We adopt Equation 19 to judge the accuracy of MBL to reach the stable phase, where AME denotes Average of Moments Estimators. When AME reaches the stable phase, the number of samples and the parameters in the dynamic model will be adjusted. Once the parameters in the dynamic model have been adjusted, the precision will be improved and AME will reach a new stable phase. The above stability, adjustment and re-stability process does not stop, until the localization obtains the desired positioning accuracy:

(19) $$\mathrm{AME}=\frac{1}{M}\;\sum_{i=1}^{M}\left(b_{i}-a_{i}\right)$$

where *M* is the number of unknown nodes, *a_i_* and *b_i_* denote the parameters of the uniform distribution of the *i*-th unknown node.

From an implementation perspective, Equation 17 will be computed in the unknown node at a certain time interval. All unknown nodes do not require send the result of (*b* – *a*) to the beacon at one time, but each unknown node sends the value when it contacts the beacon. Equation 19 will be computed in the beacon and the beacon only maintains (*b* – *a*) of the recently *M* contacted unknown nodes.

## 5. Evaluation

The key metric [16] for evaluating a localization algorithm is the accuracy of the location estimates or *localization error*. This is computed as follows:

(20) $$\mathrm{Error}=\frac{1}{M}\;\sum_{i=1}^{M}\left\|e_{i}-R_{i}\right\|$$

(21) $$M=\frac{n_{d}\;S}{{\pi r}^{2}}$$

where *M* is the number of unknown nodes and can be obtained from the Equation 21,*R_i_* denotes the real location of the *i*-th unknown node, *e_i_* denotes the location estimate of the *i*-th unknown node, and ||*e_i_* - *R_i_*|| denotes the distance between locations *e_i_* and *R_i_*. The errors shown in the simulation results are in terms of the radio range, i.e., the errors shown are computed by dividing the error in Equation 20 by the radio range of sensor node. Most parameter settings for our simulations are those used in [16,18]. Our results were obtained using sensor nodes randomly distributed in a 500 units × 500 units square field, i.e., S = 500 × 500. In our experiments, we set ideal radio range *r* = 100, the node density *n_d_* = 10, the number of samples for an unknown node *N* = 50, the parameter α = 0.1*r* in the SUD or CUD, the parameter *σ* = 0.02*r* in the ND, and the maximum speed of beacon *v_max_* = 1.0*r*. We adopt Walking GPS architecture [12] for the beacon and unknown nodes which significantly reduce the size of the code and data memory used on the sensor node. Other simulation parameters of the unknown node are based on the MicaZ sensor node. The beacon’s movement is implemented using random waypoint mobility model.

### 5.1. Dynamic Model

**Figure 3.**
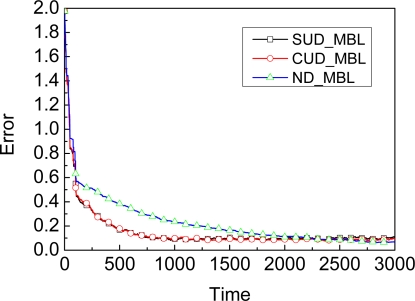
Accuracy comparison of different dynamic model.

In this section, we will evaluate the accuracy of different probability densities adopted as the dynamic model in the prediction stage for MBL. The graph in Figure 3 shows the comparison of accuracy for SUD_MBL, CUD_MBL, and ND_MBL, where SUD_MBL, CUD_MBL, and ND_MBL denote different dynamic models SUD, CUD, and ND as shown in Equation 8, 10, and 12 adopted by MBL respectively in the prediction stage.

As shown in Figure 3, on the one hand, for SUD_MBL and CUD_MBL, the curvatures of the two curves are always very close to each other, i.e., different dynamic models SUD and CUD will not affect the accuracy of MBL. On the other hand, in the initialization phase, the convergence of SUD_MBL and CUD_MBL is much faster than ND_MBL, but, in the stable phase, the accuracy of ND_MBL is a little bit higher than in SUD_MBL and CUD_MBL. In order to compare the accuracy of those different dynamic models for MBL from the quantitative point of view, we give Table 2 to show the accuracy of MBL under three different dynamic models. These experimental results are the average of 20 executions with different pseudorandom number generator seeds. In Table 2, ND_MBL shows nearly 20%–25% better accuracy when compared to the SUD_MBL and CUD_MBL. Shown in Section 3.1.3, we have known that SUD_MBL is more efficient than ND_MBL. Taking into account both the efficiency and accuracy of localization, which the dynamic model should we choose?

**Figure 4.**
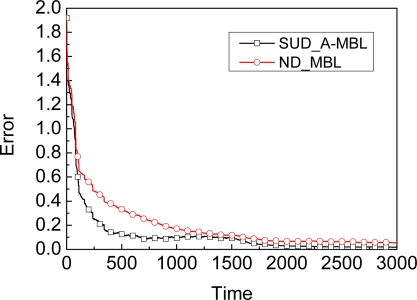
Accuracy comparison between SUD_A-MBL and ND_MBL.

**Figure 5.**
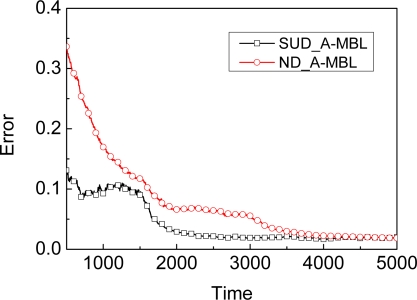
Accuracy comparison between SUD_A-MBL and ND_A-MBL.

We give another result as shown in Figure 4. Compared to the SUD_A-MBL which denotes A-MBL adopting SUD as the dynamic model, ND_MBL has no advantage due to the invariable parameter in the dynamic model, i.e., the accuracy of MBL with adapting mechanism are higher than ND_MBL with different dynamic model to improve accuracy. That is to say, SUD_A-MBL achieves higher accuracy and does not lose efficiency. Of course, if ND_MBL also adopts an adapting mechanism, then ND_MBL can achieve higher accuracy. We show the accuracy comparison results between SUD_A-MBL and ND_A-MBL in Figure 5. Though SUD_A-MBL and ND_A-MBL will reach almost the same accuracy at the end, ND_A-MBL needs more time to do so than SUD_A-MBL, i.e., the efficiency of ND_A-MBL is lower because it spends longer time achieving the stable phase of localization process.

### 5.2. Neighbors’ Observation

The threshold value *β* which mentioned in Section 3.2 depends on the number of neighbors of an unknown node. In [16], MSL uses *β* = (0.1)*^neighbor^*, where *neighbor* denotes the number of neighbors of an unknown node. In our experiments, we set *neighbor = n_d_*, i.e., *β* = (0.1)^*n*_*d*_^. To reduce computational complexity, the unknown node will use the observation from the neighbors in some interval.

**Figure 6.**
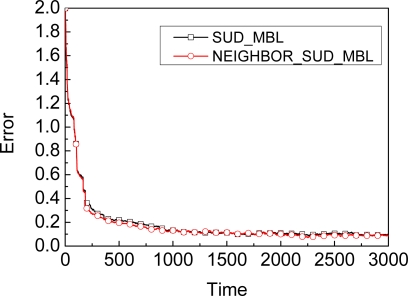
Comparison of neighbor’s impact when (Time ≥200) and (Time mod 200 = 0).

The graph in Figure 6 shows the comparison results of accuracy for SUD_MBL and NEIGHBOR_SUD_MBL when the time interval is 200, where NEIGHBOR_SUD_MBL denotes SUD_MBL relying on observation not only from the beacon, but also from its neighbors in the update stage. As shown in Figure 6, SUD_MBL and NEIGHBOR_SUD_MBL, these two curvature of the curves are always very close to each other, i.e., combined with the observation from neighbors will not improve the accuracy of MBL and have not faster convergence under these conditions.

**Figure 7.**
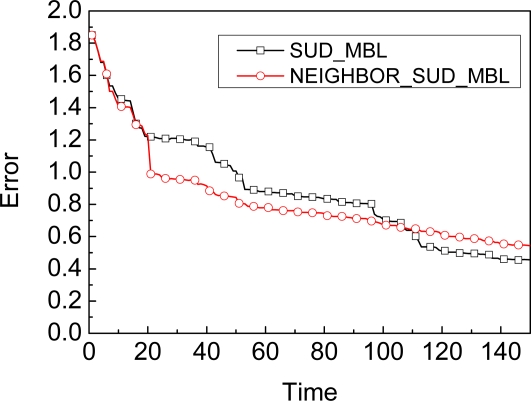
Comparison of neighbor’s impact when (Time ≥ 20) and (Time mod 20 = 0).

The graph in Figure 7 shows the comparison results of accuracy for SUD_MBL and NEIGHBOR_SUD_MBL when the time interval is 20. When the time is 20, the accuracy of NEIGHBOR_SUD_MBL is suddenly improved, just because of the impact of neighbor’s observation. After the time is larger than 106, the accuracy will not be improved by a neighbor’s observation. Obviously, NEIGHBOR_SUD_MBL is more accurate than SUD_MBL from 20 to 106.

Under the same conditions, we show the result of SUD_MBL concerning the number of observations from the mobile beacon to the unknown node. Table 3 shows the average number of observations of unknown nodes from the beacon. As shown the second column in Table 3, when the average number of observations (Number = 10) is equal to *n_d_* (*n_d_* *=* 10), SUD_MBL is still in the stabilization process (the time is 106), i.e., the samples of unknown node cannot represent the location of unknown node at this time. With the increase of the number of observations from the beacon, this number will far exceed the *n_d_*, i.e., the neighbors’ observation will not improve the accuracy of SUD_MBL.

### 5.3. SA-MBL

**Figure 8.**
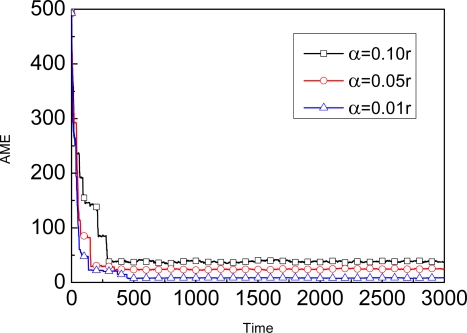
The coverage of AME in three different α.

Figure 8 shows the coverage of AME for three different *α* of SUD_MBL. Whether the parameter *α* is 0.1r, 0.05r or 0.01r, as the time goes on, the AME of the unknown node will achieve stable phase eventually. To catch the AME of the stable phase under 10 different parameters *α* for later use, we give the Table 4 to show the results. These experimental results are the average of 20 executions with different pseudorandom number generator seeds. Based on this table, SA-MBL will adjust the value *α* according to the corresponding AME. In general, when this condition *| AME_c_* - *AME_i_* | < ε is satisfied, where *AME_c_* denotes the current AME maintained in the beacon and *AME_i_* represents some value listed in the *i*-th row of the Table 4. Then, the current parameter α_c_ maintained in the unknown node will be set as α_c_ = α_i+1_. For example, when current value of AME in the beacon is nearly 34, the parameter α will be adjusted to 0.07*r*. Similarly, the number of samples *N* will be adjusted accordingly based on Table 4.

**Figure 9.**
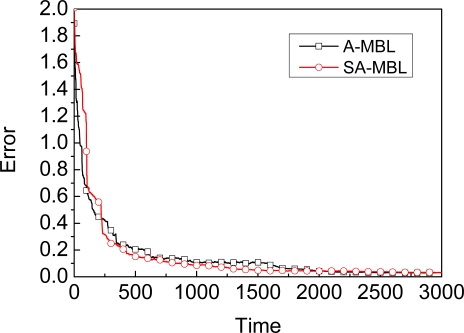
Accuracy comparison between A-MBL and SA_MBL.

**Figure 10.**
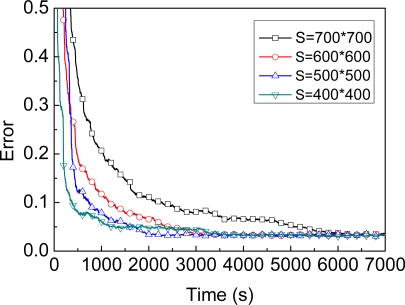
Location convergence under different deployment regions.

The graph in Figure 9 shows the comparison of accuracy between A-MBL and SA-MBL. As shown in this figre, for A_MBL and SA_MBL, the curvature of the two curves are always very close to each other, i.e., MBL with Self-Adapting mechanism will get almost the same performance with A-MBL for which obtaining predefined adjustment tables is difficult. The graph in Figure 10 shows the efficiency and accuracy comparison for SA-MBL under the same conditions except for the deployment region. In order to obtain close precision, the largest the deployment region (S = 700 × 700) will spend the longest convergence time among these experiments.

**Figure 11.**
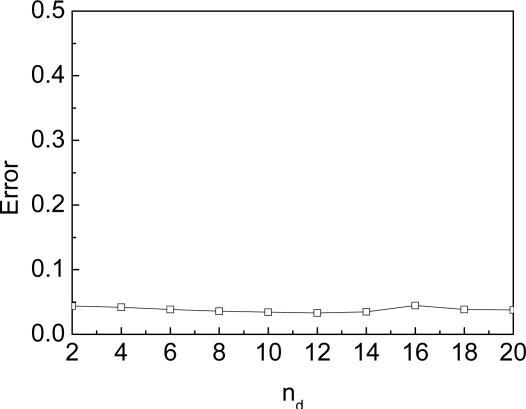
Unknown node density.

In another experiment we vary the unknown node density from 2 to 20. We set the time of beacon movement as 3,000 to achieve the stable phase. As shown in Figure 11, the unknown node density will not affect the accuracy of SA-MBL. That is to say, the adapting mechanism in SA-MBL can be unrelated to the deployment region, the location time and node density, i.e., the approach is actually self-adapting. Finally, the localization error bar in Figure 11 shows that the SA-MBL is stable.

## 6. Conclusions

In this paper, we compare the efficiency of various probability distributions in the dynamic model, and evaluate the accuracy of these approaches adopted as the dynamic model. We suggest that SUD should be adopted as the dynamic model due to achieve higher accuracy and not lose efficiency. We also conclude that the neighbors’ observation do not achieve more accuracy of MBL than expected. Finally, we propose SA-MBL which can judge the accuracy of MBL to reach the stable phase as the effect of predefined adjustment tables in A-MBL and be unrelated to the scale deployment of sensor nodes, localization time, and the speed of the beacon, etc., but just related to the unknown nodes themselves. As a result, SA-MBL can achieve more flexibility and result in almost the same performance as A-MBL. In future work, a number of practical factors will be considered, and the practical factors may affect efficiency, accuracy, and flexibility of our localization approaches.

## Figures and Tables

**Figure 12.**
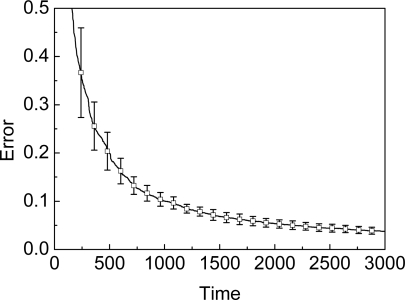
Error bar of SA-MBL.

**Table 1.** The efficiency of different algorithms.

**Probability Distribution**	**UD (Uniform Distribution)**			**ND (Normal Distribution)**	
	**SUD**	**CUD**			
Simulation Method		Accept-Reject	Polar	Box-Muller	Polar
Random Numbers	2	2.546	2	2	2.546
Trigonometric Function	0	0	2	2	0
Logarithm	0	0	0	1	1
Square Root	0	0	1	1	1
Multiplication	4	7.638	5	6	10.638
Division	0	0	0	0	1

**Table 2.** The accuracy of different dynamic model.

**Name**	**SUN_MBL**	**CUD_MBL**	**ND_MBL**
**Error**	0.097430	0.090572	0.073601

**Table 3.** The average number of observations from beacon.

**Time**	**106**	**208**	**500**	**1000**	**2000**	**3000**
**Number**	10	20	45	91	176	260

**Table 4.** Parameters for ten different values of AME.

**No.**	**α**	**N**	**AME**
1	0.10r	50	38.749791
2	0.09r	50	36.766873
3	0.08r	40	34.275935
4	0.07r	40	30.948355
5	0.06r	30	28.403102
6	0.05r	30	24.881642
7	0.04r	20	21.765849
8	0.03r	20	18.161460
9	0.02r	10	13.720697
10	0.01r	10	08.323300
